# Automatic Tissue Image Segmentation Based on Image Processing and Deep Learning

**DOI:** 10.1155/2019/2912458

**Published:** 2019-01-31

**Authors:** Zhenglun Kong, Ting Li, Junyi Luo, Shengpu Xu

**Affiliations:** ^1^Northeastern University, Boston, MA, USA; ^2^Institute of Biomedical Engineering, Chinese Academy of Medical Science and Peking Union, Tianjin 300192, China; ^3^University of Electronic Science and Technology of China, Chengdu, China

## Abstract

Image segmentation plays an important role in multimodality imaging, especially in fusion structural images offered by CT, MRI with functional images collected by optical technologies, or other novel imaging technologies. In addition, image segmentation also provides detailed structural description for quantitative visualization of treating light distribution in the human body when incorporated with 3D light transport simulation methods. Here, we first use some preprocessing methods such as wavelet denoising to extract the accurate contours of different tissues such as skull, cerebrospinal fluid (CSF), grey matter (GM), and white matter (WM) on 5 MRI head image datasets. We then realize automatic image segmentation with deep learning by using convolutional neural network. We also introduce parallel computing. Such approaches greatly reduced the processing time compared to manual and semiautomatic segmentation and are of great importance in improving the speed and accuracy as more and more samples are being learned. The segmented data of grey and white matter are counted by computer in volume, which indicates the potential of this segmentation technology in diagnosing cerebral atrophy quantitatively. We demonstrate the great potential of such image processing and deep learning-combined automatic tissue image segmentation in neurology medicine.

## 1. Introduction

**Figure 1:**
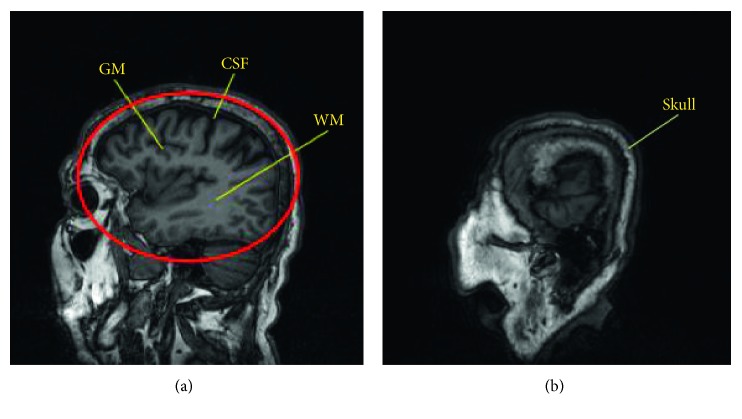
MRI images: (a) location of the 3 tissues. WM is the area where the color is light; GM is the gray boundary around the WM; CSF is the black parts inside the skull. We focus on segmenting the tissues in the red circle. (b) Cross section of a side of the brain; the tissues are hard to distinguish by eyes.

Nuclear magnetic resonance imaging gives a clear and high-resolution image of brain tissues [1]. It is a common method for clinical examination of brain diseases. The human brain structure is very complicated. Important tissues include grey matter, white matter, and cerebrospinal fluid [2] (Figure 1(a)). These tissues play a key role in memory, cognition, awareness, and language [3]. Cerebral atrophy/expansion [4, 5] and leukodystrophy [6] are serious brain dysfunction diseases that have a high incidence in infants and elderly people [7]. However, crucial tissues such as cerebrospinal fluid, grey matter, and white matter are hard to differentiate due to blurry boundaries, especially in the cross-sectional images that do not show the center of the brain, as shown in (Figure 1(b)). As a result, it is hard for doctors to analyze them separately and find the location of the disease [8]. With the popularization of image-aided medical diagnosis, computer-aided doctors can improve the efficiency of segmenting [9] the grey matter and white matter of the brain MRI. In MR imaging, different signal intensities and weighted images (T1 weighted and T2 weighted) can make the image display at different grey levels. Since the T1 brain magnetic image shows that the soft tissue is better [10], the experiment selects the brain magnetic resonance T1-W image as the experimental sample.

Many approaches have been made to segment the brain image automatically. Segmentation algorithms based on regional, texture, and histogram thresholds [11, 12] are simple but lack accuracy. Threshold is a simple but effective way to segment images. However, there are some limits regarding only using this method for segmentation. First, the grey scale of tissues may not be restricted in one range. This means that, if we simply use threshold to locate the tissues, it may fail to separate all the parts. Secondly, the threshold usually does not consider the spatial properties of an image. For example, the skull is a round structure that covers the other tissues. This can help us to determine the location of tissues and get more accurate segmentation images. As a result, threshold determination is often considered as an early stage sequential image process. Later, methods related to fuzzy c-means (FCM) [13, 14] and machine learning are introduced. The atlas-based method is also widely used for brain image segmentation [15]. It has a relatively complete system framework. However, explicit information such as intensity and spatial features is required in order to get accurate results [16]. Spatial and intensity features could be avoided by using convolutional neural networks (CNNs) [16]. Convolution neural network proposed by LeCun et al. [17] is a deep supervised learning method [18]. It has been applied in many fields and has made great success in image recognition [19, 20], speech recognition [21, 22], natural language processing, and so on. CNNs obtain the convolution weight by means of cyclic convolution and samples with a supervised training mode. The final realization is directly extracted from the original input, which is conducive to the classification features. The features in the image recognition are texture, shape, and structure.

MICCAI is a conference held every year focusing on medical image computing and computer-assisted intervention [23]. Recently, many methods related to deep learning were presented in the conference. Zhang et al. presented a 2D patch-wise convolutional neural networks (CNNs) approach to segment tissues from multimodal MR images of infants [24]. An *N *∗* N* size picture block was extracted from a given image, and the model is trained with these blocks. Then, the label was given to the correct identification class. In order to improve the performance of block training framework, multiscale CNNs used a variety of ways with different patch sizes. The outputs of these approaches were combined with the neural network, and the model was trained to give the correct label. This method in this paper did not include a pooling layer or consider the relation between the patches. Yang et al. [25] used a deep active learning framework to reduce the annotation effort. It was combined with fully convolutional network and active learning. Man et al. [26] proposed combining MRI multimodal information to extend CNNs to 3D, which was composed of multiple modes that formed 3D raw data.

In our paper, we use image enhancement, operators, and morphometry methods to extract the accurate contours of different tissues on 5 MRI head image datasets. After that, we utilize convolutional neural network to realize automatic segmentation of images with deep learning. Such approaches greatly reduced the processing time compared to the other methods. We also introduce parallel computing to further speed up the processing speed. Our work has a great potential in the medical field for diagnosing brain disease.

The rest of the paper is organized as follows. In Section 2, we describe our dataset, model, and training method. Our experiments and comparison with other methods are discussed in Section 3.  Section 4 concludes the paper.

## 2. Materials and Methods

### 2.1. Dataset

**Figure 2:**
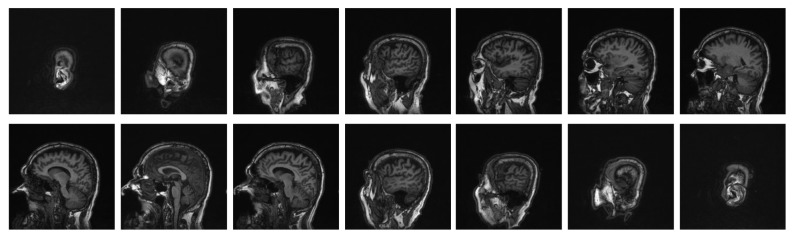
Samples of a patient's brain cross-sectional image.

Our dataset includes 5 patient's brain MRI T1-W images. For every patient, we have 160 images, with a total of 800 images. The size of the images is 256 × 256 pixels. Every pixel value in the matrix is an integer between 0 and 255.  Figure 2 shows some typical MRI of a human brain. The MRI data used to support the findings of this study are available from the corresponding author upon request.

### 2.2. Preprocessing

Due to the complicity of the brain structure, there exist many overlapping regions in each MR image. Image preprocessing can improve both the efficiency of the algorithm and the reliability of the segmentation results. Image noise reduction [27] and enhancement can make the image more conforming for viewing. By removing the bright skull, we can avoid it from affecting the accuracy of the segmentation of the brain. Image noise reduction directly affects the result of segmentation.

Wavelet domain denoising is used to transform noisy signals from time domain to wavelet domain [10] by using multiscale transformation. We removed the wavelet coefficients of noise from all scales to obtain the wavelet coefficients of signals. Finally, the signals are reconstructed by a wavelet transform. The image after noise reduction preserves the details of the original image, and the visual effect becomes clearer. The histogram equalization method is used to enhance the image of the cerebrospinal fluid, grey matter, and white matter.

**Figure 3:**
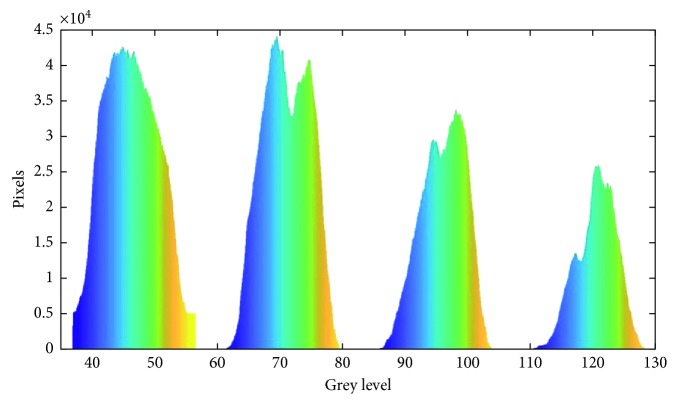
There are four peaks in the histogram. From left to right, the peaks stand for the following: cerebrospinal fluid (40–57), grey matter (61–79.8), white matter (86–110), and skull (110–130).

We collected the grey level of all 800 MRI images and generated a histogram that contains all the points, as shown in Figure 3. The result shows 4 peaks, each stands for a kind of tissue [28]. The background grey value is not shown in the figure, which is smaller than 35. From the histogram, we can remove pixels that are not in the grey level range of the GM, WM, CSF, and skull. We convert them to level 0 to reduce the noise. The histogram shows four thresholds, which stands for the four tissues. It seems that we can segment the image by only using this result. However, there are some limits. Threshold usually does not consider spatial properties of an image. For example, the shape of the skull is round and located around the other tissues. Also, the grey level of a tissue may not be restricted around one region. The grey level of GM may be in the CSF region depending on the location. Thus, the result may not be accurate by only using the threshold as an analysis measuring method.

### 2.3. Convolutional Neural Network

Convolutional neural networks (CNNs) have recently enjoyed a great success in image recognition and segmentation. The basic structure of CNNs consists of two layers. One is the feature extraction layer (C1, C3). The input of each neuron is connected to the local receptive domain of the previous layer, extracting the local feature. Once the local feature is extracted, its positional relationship between the others can be determined. The other layer is the feature mapping network layer (S2, S4). Each computing layer is composed of multiple feature maps [29]. The feature map is a flat plane; all neuron weights are equal. The feature mapping structure uses the sigmoid function [30] as the activation function of the convolution network. In addition, the number of free parameters of the network is reduced because of the weights shared by a neuron on a mapping surface. Each convolutional layer in the CNN closely follows a computing layer for local average and second extraction. This unique extraction structure reduces the feature resolution.

**Figure 4:**
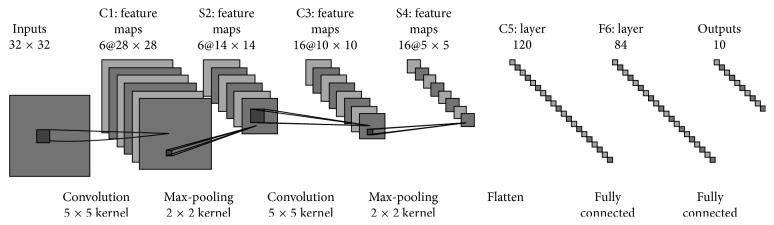
General structure of CNN. The input layer is 32* *∗* *32. The input is convoluted to six feature maps in the C1 layer by 5* *∗* *5 convolution kernel. S2 is a pooling layer with six 14* *∗* *14 features. Each unit in the feature map is connected to the 2* *∗* *2 neighborhood of the corresponding feature map in the C1 layer. The C3 layer is also a convolutional layer that uses a kernel of 5 × 5 to convolute the layer S2. The S4 layer is a pooling layer that consists of sixteen 5* *∗* *5 size feature maps. The C5 layer is a convolutional layer with 120 feature maps. The F6 layer has 84 units and is fully connected to the C5 layer. The output layer has a unit with 84 inputs.

The C1 layer (Figure 4) is a convolutional layer with six feature maps. Each neuron in the feature map is connected to the 5* *∗* *5 input. The size of the feature map is 28* *∗* *28. S2 is a pooling layer with six 14* *∗* *14 features. Each unit in the feature map is connected to the 2* *∗* *2 neighborhood of the corresponding feature map in the C1. The four inputs in each unit are added in S2 and multiplied by a trainable parameter, along with a trainable offset. The 2* *∗* *2 receptive field of each unit does not overlap, so the size of each feature map in S2 is 1/4 of the size as in C1.

The C3 layer is also a convolutional layer which uses a kernel of 5 × 5 to convolute the layer S2. The feature map has only 10 × 10 neurons but with 16 different convolution kernels. Hence, there are 16 feature maps. Each map in C3 consists of all 6 or several feature maps in S2. The reason why we do not connect each feature map of the S2 to C3 is that the incomplete connection mechanisms keep the number of connections within a reasonable range. Moreover, it destroys the symmetry of the network. Since different feature maps have different inputs, it forces them to extract different features. The S4 layer is a pooling layer that consists of sixteen 5* *∗* *5 size feature maps. Each unit in the feature map is connected to the 2* *∗* *2 neighborhood of the corresponding feature map in the C3, same as the C1 and S2. The F6 layer has 84 units and is fully connected to the C5 layer. Finally, the output layer is composed of a Euclidean radial basis function unit, each of which has a unit with 84 inputs.

The output of the convolution layer is the sum of the convolution kernel and the output of the upper layer:

(1) $$\begin{array}{c} x_{j}^{l}=f\left(\underset{i\in M_{j}}{\sum }x_{i}^{l-1}\;*\;k_{ij}^{l}+b_{j}^{l}\right), \end{array}$$

where *l* is the layer number, *k*  is the convolution kernel, *b* is the bias, *x* is the input, and *M*_*j*_ is the chosen feature map.

The number of input and output in the pooling layer are the same, while the dimension of the maps is reduced:

(2) $$\begin{array}{c} x_{j}^{l}=f\left(\beta_{j}^{l}\text{ down }\left(x_{i}^{l-1}\right)+b_{j}^{l}\right). \end{array}$$

**Figure 5:**
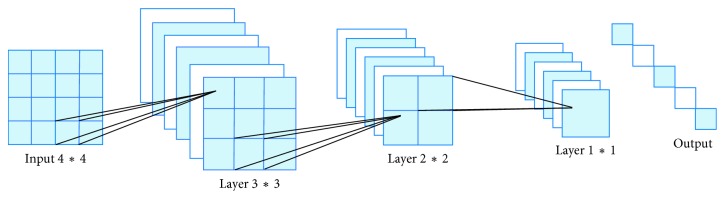
Our CNN structure.

The parameters of the CNN are set as follows (Figure 5). The neural network is divided into three layers: the input layer is many 4* *∗* *4 pixel units, the second layer is the convolution layer with 6 kernel functions of 3* *∗* *3, and the third layer is the down-sampling layer with six 2* *∗* *2 pixel units. Finally, the parameters of the stable network after training are obtained. In each layer, there are many 2D planar elements, and each 2D planar element has many independent neurons. The output has six pixels, thus extracting the deep feature data. We used Adam algorithm for the learning method. We set the initial learning rate to 0.001 and the momentum to 0.5. We used the cross entropy as the loss function. Our CNN was trained for 50 epochs, each consisting of 20 subepochs. Our batch size is 5. Training samples are randomly selected from the total 800 images: 600 images are for training, 100 images are for validation, and 100 images are for testing.

### 2.4. Parallel Computing

MPI is at the core of many supercomputing software frameworks [31]. We used a Caffe framework that adopts the MPI. The MPI enables the cluster version to optimize the data parallel to Caffe. It supports command line, Python, and MATLAB interfaces and various programming methods. The CPU information is Intel(R) Xeon(R) CPU E5-2670 0 @ 2.60 GHz. We convert the 800 matrices into one large matrix, which has the size 800* *∗* *65536. In addition, we added a row for placing the image number. Each row is an image. The first number in each row is the index of the image. The image data are put together in one matrix. Thus, the processing involves all the images at one time. We adopt the master-slave mode [32]. It includes two sets of processes: the master processor is in charge of processing the work orders [33]. The slaves execute the work that the master processor assigns.

**Figure 6:**
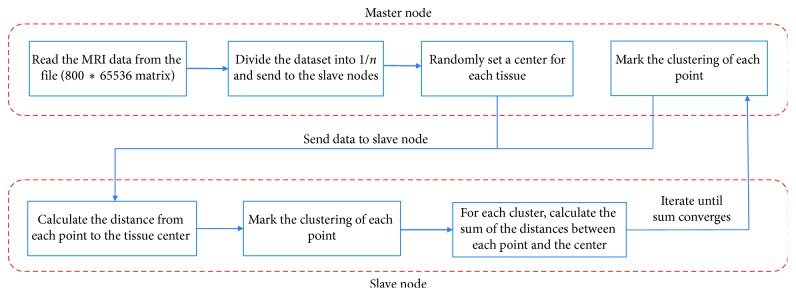
Parallel computing master-slave method. The master processor is in charge of processing the work orders. The slaves execute the work that the master processor assigns. The process repeats until the sum of the distances of all the clusters is constant.

In our work, one node acts as the master node, which is responsible for data partition and allocation. The other nodes complete the calculation of local data and return the result to the master node. As shown in Figure 6, the master node first reads the data and assigns them to the other nodes and then selects the center of each cluster. The slaves calculate the distance from each point to the center of the data block, then mark the clustering of each point, calculate the sum of the distances between all the points of each cluster to the center of the cluster, and finally return these results to the master. The cluster centers stand for the tissues in the GM, WM, CSF, and skull, which are spatial coordinates. We use the Euclidean distance to find the center of the tissue feature clusters, respectively, and set the parameter ∂ = 0.5, which takes out 50% feature points that are nearest to the feature center points to accurately characterize the quantitative characteristics of the different types of data. The master node will calculate the new center point, send to other processes, and calculate the other process from the clustering of all points to the center of the sum of the distance. The process will continue until the sum of the distances of all the clusters is constant.

## 3. Results

### 3.1. Tissue Segmentation

**Figure 7:**
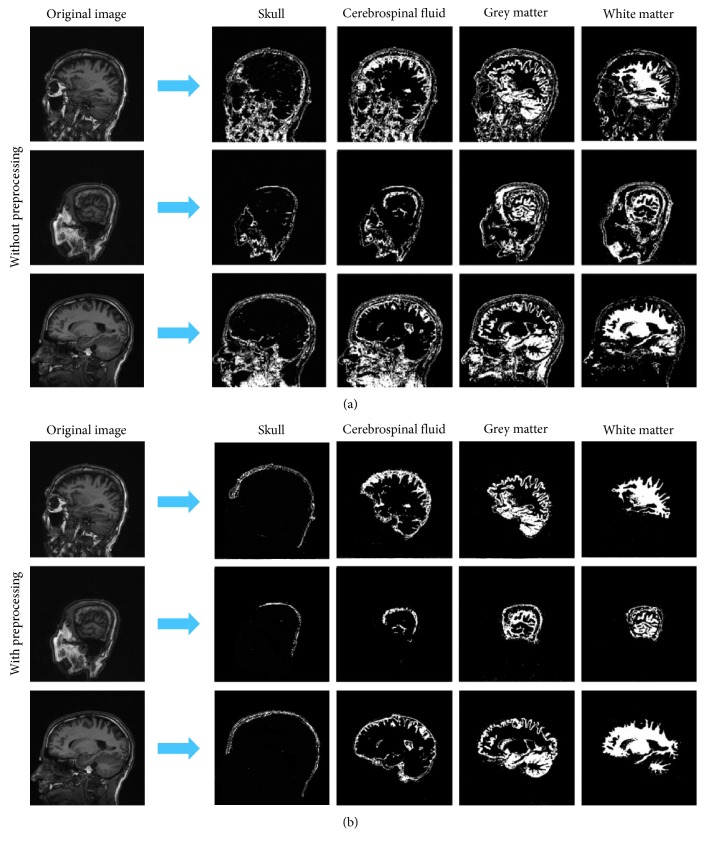
(a) Images generated without preprocessing. The first column shows the skull results; the second column shows the cerebrospinal fluid results; the third column shows the grey matter results; the fourth column shows the white matter results. (b) Images generated with preprocessing.

Our work shows some satisfying results (Figure 7(b)). The images in the left column are the original images. They show a full MRI image of the tissues of the brain and other parts such as the facial skull, muscle, and ears. After removing the other parts of the head apart from the brain that are considered noise, we successfully segment one brain image into 4 images. In each image, we set the gray value of the tissue as 255 and the background as 0.

The result of the skull shows a curved shape located on the frontier of the brain. The cerebrospinal fluid is the segment between the skull and the grey matter/white matter. The grey matter and white matter are also accurately segmented.

To test the efficiency of our method, we also did segmentation without preprocessing. As shown in Figure 7(a), the results include a large amount of noise including parts that are not brain such as the nose, eyes, and other facial structures.

### 3.2. Comparison with Visible Chinese Human (VCH)

Our result was compared pixel by pixel with the images segmented by an expert operator. To quantize our result, we calculated the percentage of each tissue in the human brain and then compared it to the visible Chinese human head (VCH) model [34]. The VCH model is a very good presentation for the anatomical structure. It is mostly used in modeling light propagation [35]. It can help calculate the volume of brain tissues because the intensity of light changes while propagating. The VCH model is developed from high-resolution cryosectional color photographs of a reference adult male [36, 37]. It includes various types of tissues from a standing frozen man body. The section precision is horizontally 0.02 cm interval, and the digital color image has a resolution of 0.01 cm per pixel, which is higher than CT and MRI [38]. Thus, it is one of the most realistic head models that contains precise cerebral cortex folding geometry [39].

**Figure 8:**
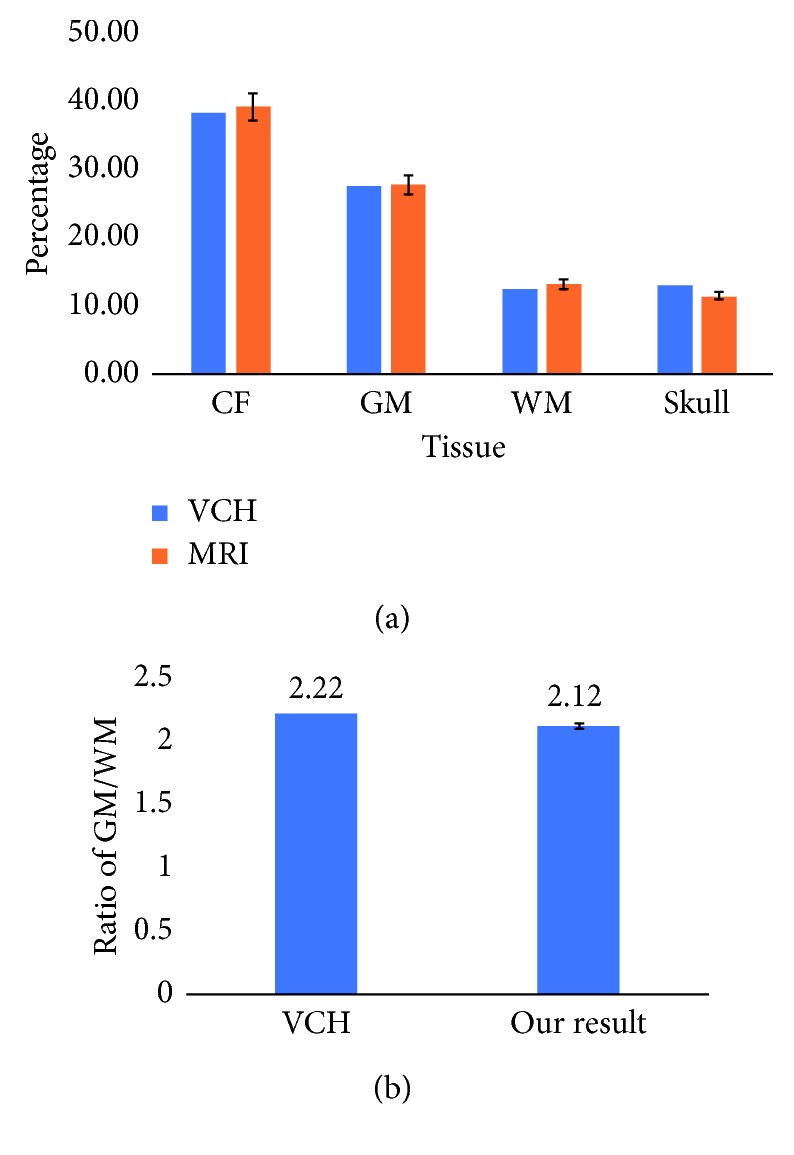
(a) A comparison of the percentage of each tissue in the brain between our result and the VCH result. (b) Ratio of grey matter and white matter.

The data are shown in Table 1. Overall, our result is quite satisfying. We manage to locate the boundary and segment the tissues accurately (Figure 8(a)). We then calculated the ratio between the grey matter and white matter and compared it to the VCH result, giving an accuracy of 95% (Figure 8(b)). This can be used in diagnosing disease such as cerebral atrophy, which is caused by grey matter or white matter reduction. There are still some deviations between our result and the ground truth. This is caused by the remaining noise and the insufficiency of the algorithm. The MRI dataset contains slit images of the brain instead of crosscut, which shows a full head rather than only the brain. As a result, more noise will be generated from the other part of the head. Taking the GM percentage, for example, some parts that are apart from the brain have the same grey value as the GM, which adds to the total percentage of the GM. For further research, we will use the dataset from BrainWeb, an online interface that provides a 3D MRI-simulated brain database [40]. It also provides a fuzzy model for users to estimate the partial volume and is a good way to verify the accuracy of our method.

For the ratio between the grey matter and white matter, we compared our result with the VCH model, along with some studies done by others. Bartlett et al. introduced an interactive segmentation (IS) method to get the volume of the GM and WM from MRI images [41]. Ge et al. investigated the effects of age and sex on the GM and WM volumes by using volumetric MR imaging in healthy adults [42]. The average results are also shown in Table 2.

We also calculated the Jaccard index for each tissue, as shown in Table 3.

Our ground truth is the visible Chinese human. We calculated the coefficient between our result and the ground truth. Our method performs well on segmenting CF, GM, and WM, outperforming the methods from Hasanzadeh et al. [44] and Luo et al. [26].

### 3.3. Comparison with FreeSurfer

**Figure 9:**
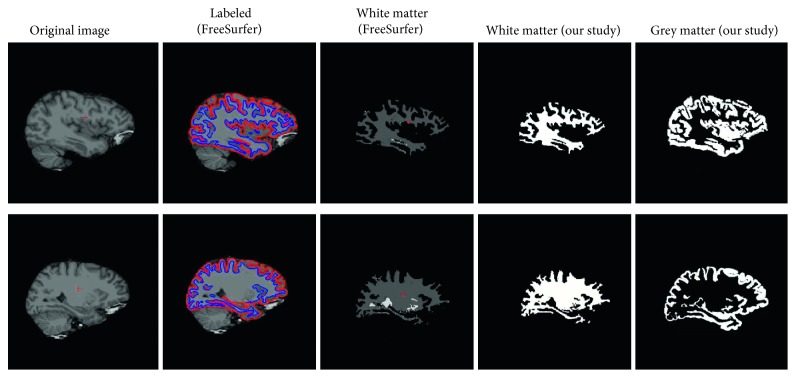
Comparison between our method and FreeSurfer.

For further experiment, we compared our result with FreeSurfer. FreeSurfer is the software built for analyzing and visualizing the structural and functional neuroimaging data from cross-sectional or longitudinal studies [43]. The results are shown in Figure 9. Our method has many advantages. FreeSurfer can only locate the WM and GM from an MRI image. Also, it cannot show separate results, except the WM. The image of the WM has many defects: it has many miss-labeling and error-labeling, such as white spots in the image. Our method performs better than this software; we can segment every tissue clearly and also display the results separately.

### 3.4. Runtime

**Figure 10:**
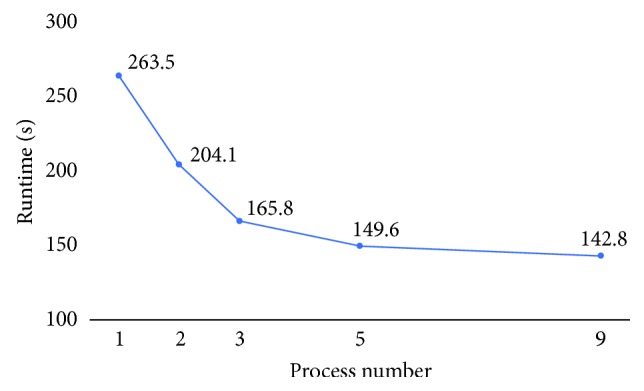
Runtime under different numbers of processes.

By introducing parallel computing, we managed to reduce the runtime. We adopt the master-slave mode. One node acts as the master node, which is responsible for data partition and allocation. The other nodes complete the calculation of local data and return the result to the master node. This is important when facing large data. Our result is not significant due to the limitation of the data size. The larger the dataset, the better the result obtained.  Figure 10 shows a visualized result of the tendency of the runtime.

## 4. Discussion and Conclusion

We used image enhancement, operators, and morphometry methods to extract the accurate contours of four tissues: the skull, cerebrospinal fluid (CSF), grey matter (GM), and white matter (WM) on 5 MRI head image datasets. Then, we realize automatic image segmentation with deep learning by using the convolutional neural network. Approaches such as regional, texture, and histogram threshold algorithms and fuzzy c-means (FCM) have limitations in processing time, accuracy, and datasets [16]. In our paper, the percentage of each tissue is calculated, which can be used as a criterion when diagnosing diseases such as cerebral atrophy, often caused by the grey matter or white matter reduction [8]. We also used parallel computing to reduce the runtime.

In our method, the preprocessing step improved the efficiency of the algorithm and the reliability of the segmentation result. Wavelet domain denoising is used to transform noisy signal from time domain to wavelet domain [10] by using multiscale transformation. We removed the wavelet coefficients of noise from all scales to obtain the wavelet coefficients of signals. This way, the efficiency of the algorithm and the reliability of the segmentation result were improved.

We introduced convolutional neural networks to our work. The CNN consists of two types of layers, a convolution layer and a pooling layer. Multiple feature maps are generated from the convolution layer after convolution. After that, the pixels of each group in the feature map are modified by adding weighted values and offset, along with a sigmoid function to get the feature map in the pooling layer. With multiple convolution and pooling layers, we were able to get more accurate results in less amount of time compared to manual and semiautomatic segmentation. We also used parallel computing to further reduce the runtime of the process. We adopted the master-slave mode by setting one node as the master node, which is responsible for data partition and allocation, along with other nodes to complete the calculation of local data and return the result to the master node.

We compared our results with the data from the visible Chinese human (VCH) head model [34]. The VCH model gives a very good presentation of the anatomical structure and is mainly used in modeling light propagation [35]. The data are collected from high-resolution cryosectional color photographs of a reference adult male. It includes various types of tissues from a standing frozen man body, which includes precise cerebral cortex folding geometry [37]. It gave us an average percentage of each tissue (skull, cerebrospinal fluid, grey matter, and white matter) in the brain. The deviation of our result was less than 2.21%. Another important index is the ratio of GM to WM, which helps in evaluating certain pathological changes of brain. Our result is also very satisfying, with an accuracy rate up to 95%. Our dataset includes 5 human brains with 160 images each. Therefore, the results are convincing. Our work concentrates on the total percentage between the tissues; we did not compare the accuracy of our boundary with other studies. For further research, we will focus more on the comparison between the boundary and use dataset from BrainWeb, which is an online interface that provides 3D MRI-simulated brain database. It provides a fuzzy model for users to estimate the partial volume and is a good way to verify the accuracy of our method.

Our research has some limitations. First of all, due to limited time, our dataset is not very large. We will increase the quantity and also the variety of the samples, including different races such as black, white, and yellow people in the future. We also need to collect samples from different ages, ranging from the infant, the juvenile, to the elderly. Currently, our dataset includes only adults. With a bigger dataset, we can classify the samples into age, gender, race, and more. We hope to set up a criterion of judgment for medical diagnosing. Researchers and doctors can compare the brain data of a patient with our data and confirm the abnormal proportion of tissues in the brain and further diagnose what disease the patient has.

To conclude, we presented a method to successfully segment the brain tissues from MRI using the convolutional neural network. The percentage results are very close to the average human brain data generated by the VCH model. This is a breakthrough since artificial intelligence and machine learning have become more and more widely used in research. By introducing deep learning into the therapeutic field, the speed and accuracy can be improved. This is because machines can automatically analyze the data, which can be much faster and accurate than the manual and semiautomatic analysis. For future work, we can visualize the contours of the borders of different tissues in 3D so that it can be integrated with optical simulation software such as MCVM for low-level light therapy. Our work has a great potential in the medical field, and we hope that our technique can be a criterion of judgment for diagnosing.

## Figures and Tables

**Table 1:** A comparison of the percentage of each tissue in the brain.

Type	VCH (%)	MRI (%)
CSF	37.65	38.54 ± 2.21
GM	27.08	27.33 ± 1.47
WM	12.35	12.95 ± 0.94
Skull	12.81	11.32 ± 1.33

**Table 2:** The ratio between the grey matter and white matter.

Type	GM/WM
MRI	2.12
VCH	2.22
IS	2.03
Ge	1.5

**Table 3:** Jaccard index of four tissues.

Type	Jaccard index
CSF	0.9431
GM	0.9020
WM	0.9142
Skull	0.8799

## Data Availability

The MRI data used to support the findings of this study are available from the corresponding author upon request.
